# Differential Impact of Hyperglycemia in Critically Ill Patients: Significance in Acute Myocardial Infarction but Not in Sepsis?

**DOI:** 10.3390/ijms17091586

**Published:** 2016-09-21

**Authors:** Bernhard Wernly, Michael Lichtenauer, Marcus Franz, Bjoern Kabisch, Johanna Muessig, Maryna Masyuk, Malte Kelm, Uta C. Hoppe, Christian Jung

**Affiliations:** 1 Clinic of Internal Medicine II, Department of Cardiology, Paracelsus Medical University of Salzburg, Salzburg A-5020, Austria; b.wernly@salk.at (B.W.); m.lichtenauer@salk.at (M.L.); u.hoppe@salk.at (U.C.H.); 2 Clinic of Internal Medicine I, Department of Cardiology, Jena University Hospital, Thuringia 07743, Germany; marcus.franz@med.uni-jena.de (M.F.); bjoern.kabisch@med.uni-jena.de (B.K.); 3 Division of Cardiology, Pulmonology, and Vascular Medicine, Medical Faculty, University Duesseldorf, Düsseldorf 40225, Germany; johanna.muessig@med.uni-duesseldorf.de (J.M.); maryna.masyuk@med.uni-duesseldorf.de (M.M.); malte.kelm@med.uni-duesseldorf.de (M.K.)

**Keywords:** hyperglycemia, sepsis, myocardial infarction, critically ill, stress hyperglycemia, diabetes, prediabetes

## Abstract

Hyperglycemia is a common condition in critically ill patients admitted to an intensive care unit (ICU). These patients represent an inhomogeneous collective and hyperglycemia might need different evaluation depending on the underlying disorder. To elucidate this, we investigated and compared associations of severe hyperglycemia (>200 mg/dL) and mortality in patients admitted to an ICU for acute myocardial infarction (AMI) or sepsis as the two most frequent admission diagnoses. From 2006 to 2009, 2551 patients 69 (58–77) years; 1544 male; 337 patients suffering from type 2 diabetes (T2DM)) who were admitted because of either AMI or sepsis to an ICU in a tertiary care hospital were investigated retrospectively. Follow-up of patients was performed between May 2013 and November 2013. In a Cox regression analysis, maximum glucose concentration at the day of admission was associated with mortality in the overall cohort (HR = 1.006, 95% CI: 1.004–1.009; *p* < 0.001) and in patients suffering from myocardial infarction (HR = 1.101, 95% CI: 1.075–1.127; *p* < 0.001) but only in trend in patients admitted to an ICU for sepsis (HR = 1.030, 95% CI: 0.998–1.062; *p* = 0.07). Severe hyperglycemia was associated with adverse intra-ICU mortality in the overall cohort (23% vs. 13%; *p* < 0.001) and patients admitted for AMI (15% vs. 5%; *p* < 0.001) but not for septic patients (39% vs. 40%; *p* = 0.48). A medical history of type 2 diabetes (*n* = 337; 13%) was not associated with increased intra-ICU mortality (15% vs. 15%; *p* = 0.93) but in patients with severe hyperglycemia and/or a known medical history of type 2 diabetes considered in combination, an increased mortality in AMI patients (intra-ICU 5% vs. 13%; *p* < 0.001) but not in septic patients (intra-ICU 38% vs. 41%; *p* = 0.53) could be evidenced. The presence of hyperglycemia in critically ill patients has differential impact within the different etiological groups. Hyperglycemia in AMI patients might identify a sicker patient collective suffering from pre-diabetes or undiagnosed diabetes with its’ known adverse consequences, especially in the long-term. Hyperglycemia in sepsis might be considered as adaptive survival mechanism to hypo-perfusion and consecutive lack of glucose in peripheral cells. AMI patients with hyperglycemic derailment during an ICU-stay should be closely followed-up and extensively screened for diabetes to improve patients’ outcome.

## 1. Introduction

Hyperglycemia is a common condition in critically ill patients. Transient hyperglycemia in this context is usually referred to as “stress hyperglycemia” [1,2,3]: In contrast to hyperglycemia due to type 2 diabetes mellitus, stress hyperglycemia is primarily caused by hepatic gluconeogenesis and glycogenolysis [4]. Patients admitted to an intensive care unit (ICU) represent an inhomogeneous collective and hyperglycemia might need a differential evaluation depending on the underlying disorder.

Hyperglycemic derailment is known to be associated with adverse outcome and increased mortality in patients suffering from an acute myocardial infarction (AMI) [5]. In these patients there is an ongoing debate whether increased glucose concentration constitutes an independent risk factor or depicts only a severity parameter of illness in certain circumstances. Of note, the association between stress hyperglycemia and mortality in AMI patients is at least more pronounced in non-diabetic patients, and it was speculated that patients with pre-existing diabetes mellitus undergo cellular adaptation to hyperglycemia as reactive oxygen species production by the mitochondria is reduced [6,7].

In septic patients severe hyperglycemia (blood glucose >200 mg/dL) but not mild hyperglycemia (141–199 mg/dL) was recently reported to be associated with increased mortality [8]. Up to a certain degree, stress hyperglycemia is thought to be adaptive to hemodynamic changes in patients suffering from sepsis [9]: Sufficient glucose supply is essential for all cells. As sepsis leads to hypo-perfusion and glucose uptake depends on a concentration gradient, an increase in blood glucose levels is a physiologic and necessary response mechanism to hypo-perfusion [10]. Whereas in the early 2000s tight glucose control in septic patients was thought to be beneficial, it could be shown in large, prospective trials that intensive glucose control is even detrimental for these patients [11,12,13]. Therefore, in septic patients, stress hyperglycemia is considered a beneficial response of the organism and only liberal management of high glucose, avoiding glucose concentrations leading to fluid shifts by serum osmolality changes, is recommended [14]. It is well known that patients suffering from diabetes are at increased risk for infection and sepsis due to humoral defects and impaired host response [15,16,17]. Even though, and in contrast to AMI patients, septic patients with known diabetes mellitus do not reveal increased mortality as reported by van Vught et al. and Esper et al. [8,18].

Both, septic and AMI patients are frequently admitted to ICUs, but a detailed assessment of these collectives is needed. We therefore compared associations of severe hyperglycemia and mortality in ICU patients suffering from either AMI or sepsis with and without pre-diagnosed diabetes mellitus.

## 2. Results

In total, 2551 patients were investigated. Among these, 572 were admitted to the ICU due to sepsis and 1979 patients because of an AMI. Mean follow-up time was 2135 ± 45 days. Baseline characteristics are shown in Table 1. Patients admitted for sepsis were of similar age (68 (58–77) vs. 69 (59–77) years, *p* = 0.18) but were clinically sicker as mirrored by both, higher SAPS2 (54 ± 20 vs. 33 ± 16; *p* < 0.001) and APACHE (26 ± 8 vs. 16 ± 8; *p* < 0.001) scores compared to patients admitted for AMI. Further, heart rate was higher in septic patients (118 ± 18 vs. 91 ± 20 bpm (beats per minute); *p* < 0.001) and they evidenced more pronounced laboratory organ failure, higher white blood cell count (16.5 ± 14.1 vs. 11.2 ± 4.7; *p* < 0.001) and lactate concentration (4.4 ± 4.9 vs. 2.1 ± 3.3 mmol/L; *p* < 0.001) on the admission day as shown in Table 1.

As continuous variable, maximum glucose concentration on the day of admission was associated in a Cox regression analysis with mortality in the overall cohort (HR = 1.006, 95% CI: 1.004–1.009; *p* < 0.001) and in patients suffering from AMI (HR = 1.101, 95% CI: 1.075–1.127; *p* < 0.001) but only in trend in patients admitted to an ICU for sepsis (HR 1.030, 95% CI: 0.998–1.062; *p* = 0.07) (Table 2).

**Figure 1:**
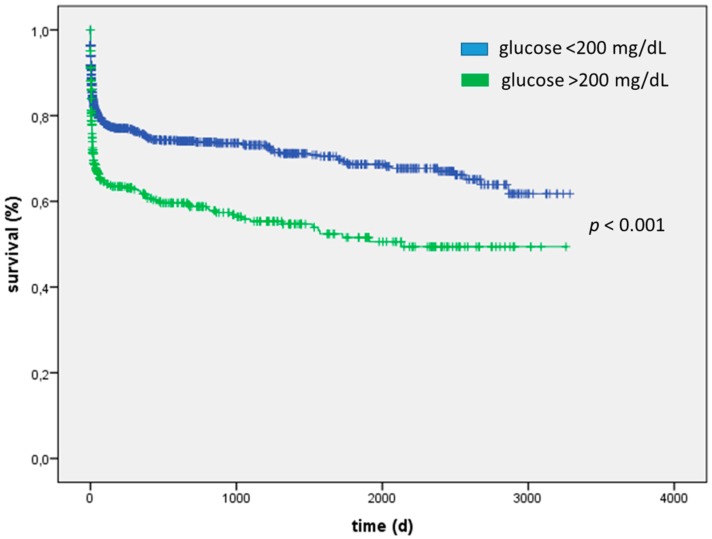
In the overall population severe hyperglycemia (>200 mg/dL) was associated with increased mortality in the long-term (HR 1.74, 95% CI: 1.44–2.09; *p* < 0.001).

To further analyze effects of severe hyperglycemia (i.e., a glucose concentration above 200 mg/dL) we split our cohort in two groups based on this cut-off. In the overall cohort, patients with severe hyperglycemia were older (71 (63–78) vs. 69 (57–77) years; *p* < 0.001), more obese (BMI 28 ± 5 vs. 27 ± 4; *p* < 0.01) and sicker (APACHE2 score 24 ± 10 vs. 19 ± 9; *p* < 0.01; SAPS2 score 47 ± 20 vs. 39 ± 20; *p* < 0.01). Patients suffering from severe hyperglycemia evidenced higher lactate concentrations (4.1 ± 5.8 vs. 2.4 ± 3.1; *p* < 0.001), higher heart rate (107 ± 24 vs. 98 ± 23 bpm; *p* < 0.001) and more pronounced laboratory multi-organ failure as shown in Table 3. In our overall population, severe hyperglycemia (>200 mg/dL) was associated with increased mortality both intra-ICU (23% vs. 13%; *p* < 0.001) and long-term (HR = 1.74, 95% CI: 1.44–2.09; *p* < 0.001) (Table 4 and Figure 1).

**Figure 2:**
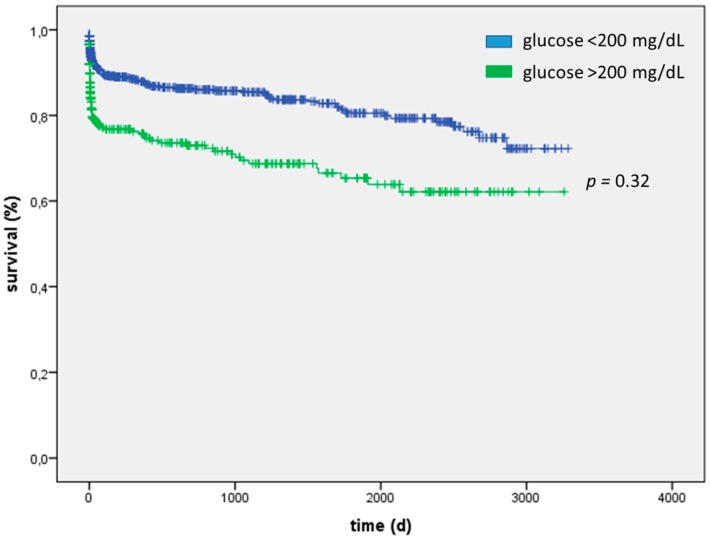
Patients admitted for sepsis and suffering from severe hyperglycemia did not evidence increased mortality in the long-term (HR 1.13, 95% CI: 0.89–1.44; *p* = 0.32).

For patients admitted for sepsis, severe hyperglycemia was not associated with increased mortality, neither short-term (40% vs. 39%; *p* = 0.5) nor long-term (HR = 1.13, 95% CI: 0.89–1.44; *p* = 0.32) (Table 4 and Figure 2). AUC for blood glucose concentration was low (AUC 0.52, 95% CI: 0.49–0.56). Interestingly, in sepsis, severe hyperglycemic patients were of equal age compared with those without severe hyperglycemia (68 (59–77) vs. 68 (55–76) years; *p* = 0.21) and whereas APACHE2 score was higher (28 ± 8 vs. 26 ± 8; *p* = 0.02) SAPS2 scores (55 ± 20 vs. 53 ± 20; *p* = 0.25) did not differ between the groups indicating a clinically similarly sick collective. Further biomarkers of renal (creatinine 184 (114–317) vs. 180 (112–292) µmol/L; *p* = 0.46) and liver failure (ALAT 0.7 (0.4–1.2) vs. 0.7 (0.3–1.4) µmol/(l*s), *p* = 0.69; ASAT 1.1 (0.5–2.9) vs. 1.0 (0.4–2.5) µmol/(l*s), *p* = 0.34) and blood lactate concentration (4.8 ± 5.3 vs. 4.1 ± 4.8 mmol/L; *p* = 0.17) and heart rate (119 ± 23 vs. 118 ± 22 bpm; *p* = 0.65) were similar (Table 5).

**Figure 3:**
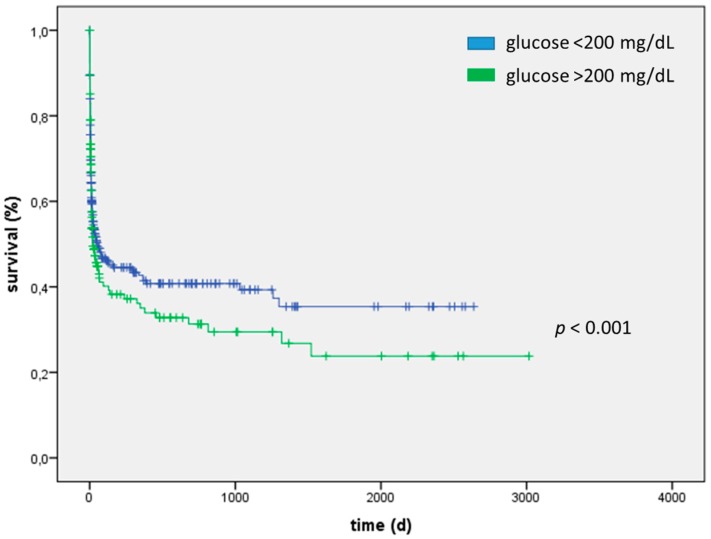
Myocardial infarction patients with severe hyperglycemia at admission day evidenced a significantly increased mortality in the long-term (HR 2.19, 95% CI: 1.66–2.89; *p* < 0.001).

Myocardial inarction patients with severe hyperglycemia at admission day evidenced a significantly increased mortality both short-term (15%; vs. 5% *p* < 0.001) and long-term (HR = 2.19, 95% CI: 1.66–2.89; *p* < 0.001) (Table 4 and Figure 3). ROC analysis for blood glucose concentration was performed for patients suffering from AMI (AUC 0.70, 95% CI: 0.67–0.72). Myocardial infarction patients with severe hyperglycemia were older (73 (65–79) vs. 68 (57–77) years; *p* < 0.001), more obese (BMI 29 ± 5 vs. 28 ± 4; *p* < 0.001) and clinically sicker (APACHE2 score 21 ± 9 vs. 16 ± 8; *p* < 0.001; SAPS2 score 41 ± 18 vs. 32 ± 16; *p* < 0.001). Blood lactate concentration (3.7 ± 6.1 vs. 1.8 ± 1.9 mmol/L; *p* < 0.001), heart rate (101 ± 22 vs. 92 ± 20 bpm; *p* < 0.001) and white blood cell count (13.2 ± 4.9 vs. 11.2 ± 5.2 G/L; *p* < 0.001) and markers of multi-organ failure were significantly higher in patients with severe hyperglycemia as shown in Table 6. Of note, patients suffering from severe hyperglycemia were more likely to have pre-diagnosed type 2 diabetes (29% vs. 10%; *p* < 0.001).

A medical history of type 2 diabetes (*n* = 337; 13%) was not associated with increased mortality: Neither intra-ICU (15% vs. 15%; *p* = 0.93) nor long term (HR = 1.140, 95% CI: 0.91–1.44; *p* = 0.26) in the overall cohort, nor in any of the sub-cohorts of AMI patients (HR = 0.82, 95% CI: 0.49–1.38; *p* = 0.53) and septic patients (HR = 1.29, 95% CI: 0.79–2.09; *p* = 0.32), although diabetic patients had higher blood glucose concentrations (213 ± 76 vs. 175 ± 70 mg/dL; *p* < 0.001), were older (72 (65–78) vs. 68 (57–77) years; *p* < 0.001), more obese (BMI 29 ± 4 vs. 28 ± 5; *p* < 0.001) and clinically sicker (APACHE2 score 22 ± 10 vs. 20 ± 10; *p* = 0.02; SAPS2 score 43 ± 21 vs. 40 ± 20; *p* = 0.06) (Table 7).

Of note, those patients with severe hyperglycemia-indicating at least an increased risk for diabetes- and/or a known medical history of type 2 diabetes taken together, evidenced significantly increased short-term (22% vs. 13%; *p* < 0.001) and long-term mortality (HR = 1.76, 95% CI: 1.47–2.11; *p* < 0.001) in the overall cohort, as well as in the AMI sub-group (intra-ICU 13% vs. 5%; *p* < 0.001) long-term HR = 2.13, 95% CI: 1.61–2.80; *p* < 0.001), but not in septic patients (intra-ICU 38% vs. 41%; *p* = 0.53; long term HR = 1.24, 95% CI: 0.98–1.57; *p* = 0.08).

We further investigated the role of hypoglycemia on the day of admission. In AMI patients 1.2% and in septic patients 5.4% suffered from hypoglycemia on admission day. We investigated associations of hypoglycemia, defined as glucose concentration below 50 mg/dL, with mortality. In patients suffering from sepsis hypoglycemia was associated with both long-term (HR = 2.39, 95% CI: 1.51–3.77; *p* < 0.001) and intra-ICU (68% vs. 38%; *p* = 0.002). In AMI patients hypoglycemia was associated with adverse long-term outcome (HR = 3.12, 95% CI: 1.38–7.03; *p* = 0.006) and with intra-ICU mortality at least in trend (19% vs. 8%; *p* = 0.12). To exclude an effect of hypoglycemia on the associations between severe hyperglycemia and outcome we excluded patients suffering from hypoglycemia: In septic patients long term mortality (HR = 1.15, 95% CI: 0.90–1.48; *p* = 0.27) and short-term mortality (39% vs. 38%; *p* = 0.77) were not associated with severe hyperglycemia whereas in patients suffering from AMI severe hyperglycemia was associated with both short-term (14% vs. 5%; *p* < 0.001) and long-term mortality (HR = 2.19, 95% CI: 1.65–2.90; *p* < 0.001).

## 3. Discussion

Severe hyperglycemia (blood glucose concentration >200 mg/dL) was associated with adverse outcome in the overall cohort and in patients suffering from myocardial infarction, but not in septic patients. We therefore think that hyperglycemia should be considered and handled differently in those patient collectives.

It has been proven recently that even a single peak of hyperglycemia can exaggerate oxidative stress, induce cell apoptosis as well as endothelial dysfunction and stimulate coagulation and platelet aggregation [19,20,21,22,23]. We therefore decided to report and investigate effects of the maximum blood glucose concentration on the day of admission to ICU.

Hyperglycemia is well known to be associated with adverse outcomes as well as reduced left ventricular (LV) function in AMI patients [24,25]. It is not clear until now, to what extent these associations are causal and in which way hyperglycemia itself contributes to adverse outcome. Furthermore, hyperglycemia is known to be associated with ischemia-reperfusion injury, increased infarct size in an animal model and tight glycemic control was reported to increase regenerative potential of infarcted myocardium [26,27,28]. In our study, AMI patients exhibiting severe hyperglycemia had significantly elevated SAPS2 and APACHE scores compared to those without severe hyperglycemia (APACHE2 score 21 ± 9 vs. 16 ± 8; *p* < 0.001; SAPS2 score 41 ± 18 vs. 32 ± 16; *p* < 0.001) indicating sicker patients in which tissue perfusion is impaired. This finding could be interpreted as evidence for the hypothesis that glucose concentration primarily mirrors illness severity. An investigation of a possible causative relationship between hyperglycemia and adverse outcome is beyond the scope of this study: further, prospective studies and experimental efforts are needed.

In septic patients, maximum glucose concentration on the day of admission was only in trend associated with mortality. Even severe hyperglycemia was not associated with adverse outcome in those patients. In accordance, patients suffering from sepsis and severe hyperglycemia were of similar age compared to septic patients with a blood glucose concentration <200 mg/dL, and did not evidence higher blood lactate concentrations, increased laboratory markers of multi-organ failure and SAPS2. We think this further supports the notion that hyperglycemia in sepsis, i.e., stress hyperglycemia, should not be considered harmful, as stated before by Tiruvoipati et al. [9]. In this condition, hyperglycemia should primarily be seen as an adaptive mechanism to hypo-perfusion to ensure sufficient glucose supply for peripheral cells by facilitating glucose uptake–a passive mechanism–by increasing blood glucose concentration [10]. Lesur et al. postulated that the stress response of septic patients is different to non-septic ICU patients’ levels [29]. In addition, this would be in accordance to prospective studies, e.g., NICE-SUGAR, which could even show an adverse outcome for tight glucose control in septic patients [11].

For blood glucose concentration a U-shaped relationship with outcome was shown in critically ill in several previous studies [30,31,32]. In our cohort hypoglycemia was associated with adverse outcome for both septic and AMI patients. Still, after exclusion of patients suffering from hypoglycemia the associations we reported in this study between severe hyperglycemia and sepsis or AMI, remained unaltered.

In our study, cohort a medical history of type 2 diabetes was not associated with increased mortality neither intra-ICU nor long both in AMI and septic patients. This goes in line with the findings of Wang and co-workers who could show that although diabetes leads to contractile and metabolic abnormalities during normoxia, there is no association between diabetes and increased susceptibility to injury due to ischemia [33]. On the other hand, whereas in short term optimal intensive care treatment might outplay possible detrimental effects of diabetes, in the long-term we would expect adverse outcome in patients suffering from diabetes mellitus. Only 13% of our cohort, representing a typical cohort of medical ICU-patients, was pre-diagnosed with diabetes. We therefore speculate that the neutral effect of pre-diagnosed diabetes mellitus in the long-term might be due to under-diagnosis of type 2 diabetes in our cohort, as prevalence in patients aged 65 and older is known to be as high as 25% (and rising) [34]. Due to the retrospective design of our study, we could not further elucidate this hypothesis as neither oral glucose tolerance test results nor Hba1c levels were available for analysis. This speculation is in contrast to findings of Ishihara et al. who could show—for their cohort of AMI patients—that patients with stress hyperglycemia did not show increased prevalence of diabetes at the time of presentation by means of oral glucose tolerance test (OGTT) [35]. Of note, OGTT might underdiagnose diabetes compared to Hba1c and stress hyperglycemia might still be associated with higher risk for developing diabetes [36,37].

As others reported that patients evidencing stress hyperglycemia are known to be at higher risk for impaired fasting glucose and developing diabetes, we aimed to find out if patients with a known medical history of diabetes and/or severe hyperglycemia on the admission day had adverse outcome [38,39]: This was true for the overall cohort and AMI patients (intra-ICU mortality 13% vs. 5%; *p* < 0.001; long-term mortality HR = 2.13, 95% CI: 1.61–2.80; *p* < 0.001) but could not be shown for septic patients (intra-ICU mortality 41% vs. 38%; *p* = 0.53; long term mortality HR = 1.24, 95% CI: 0.98–1.57; *p* = 0.08). This of course does not proof a relationship of stress hyperglycemia and diabetes but we strongly believe that this further under-strikes a primarily beneficial role of hyperglycemia in septic patients. On the other hand, in patients suffering from AMI, severe hyperglycemia might mirror both the severity of the acute event, as well as the patients’ comorbidities, such as diabetes, and therefore identify patients at high risk for death.

## 4. Methods

From 2006 to 2009, 2528 patients admitted to the Medical ICU at the Jena University Hospital because of either myocardial infarction (*n* = 1979) or sepsis (*n* = 549) were investigated retrospectively. Diagnosis and treatment of AMI was done according to ESC guidelines [40,41]. All patients diagnosed with AMI were admitted to ICU with exception of patients provided palliative care. Sepsis was defined as systemic inflammatory response syndrome accompanied by either microbiologically confirmed or clinically suspected infection [42,43].

Follow-up of patients was performed between May 2013 and November 2013. The primary endpoint of the study was death of any cause. Data on mortality were collected upon review of electronic in-hospital medical records or phone interviews and could be collected in 97.5% of patients. The study was approved by the local ethics committee of the Medical Faculty of the Friedrich Schiller University of Jena.

### 4.1. Laboratory Analysis

Blood samples were taken according to standard care. Laboratory parameters were obtained from the Department of Clinical Chemistry and Laboratory Medicine at the Jena University Hospital. Some laboratory values were measured repeatedly on the day of admission and we report the maximum value unless stated differently. Blood glucose concentration was measured in venous blood.

### 4.2. Calculation of SAPS2 and APACHE Score

Initial Simplified Acute Physiology Score II (SAPS2) and Acute Physiology And Chronic Health Evaluation (APACHE) scores were calculated by the treating physician within 24 h after admission as reported before [44,45].

### 4.3. Statistical Analysis

Statistical analysis was performed using SPSS (IBM Corp. Released 2013. IBM SPSS Statistics for Windows, Version 22.0. Armonk, NY, USA: IBM Corp.). Normally distributed data is given in mean ± standard deviation and compared by student’s *t*-test. Non-normally distributed data is given as median ± inter-quartile-range and compared by Mann-Whitney-*U*-Test. Chi-square test was applied to calculate differences between groups. Cox regression analysis was used to compare and Kaplan-Meier curve was used to depict survival data. ROC analysis was performed and area under the curve (AUC) was calculated.

## 5. Conclusions

In conclusion, we speculate about a Janus-faced role of severe hyperglycemia in critically ill patients: Whereas severe hyperglycemia in AMI patients might mirror and identify a sicker patient collective probably suffering from pre-diabetes or even undiagnosed diabetes, with its’ known adverse consequences on outcome, hyperglycemia in sepsis should primarily be seen as adaptive mechanism to impaired hemodynamics. Our study adds a comprehensive comparison of the association of severe hyperglycemia and mortality in AMI versus septic patients.

In our opinion, in the short-term, hyperglycemia should still be treated only liberally in both patients suffering from AMI as well as from sepsis, and glucose control should primarily focus on avoidance of fluid shifts due to changes in plasma osmolality. However, AMI patients with hyperglycemic derailment during an ICU-stay might be at higher risk of diabetes and consequent death. We therefore would like to suggest that these patients should be closely followed-up and extensively screened for diabetes and its complications to ensure optimal long-term outcome for all our patients.

## 6. Limitations

Our study is of single-center and retrospective nature. Regarding diagnosis of diabetes, we have neither Hba1c nor OGTT values but data from medical history. Moreover, there were no data available on either the pathogenesis of sepsis/AMI in our patients collective nor the cause of death. Patients were treated according to the treating physician’s decisions following international guidelines, but we have no data about specific antibiotic regime, parenteral/enteral nutrition and other medical interventions. Especially, we have no specific data about blood glucose management as this too was handled according to the treating physician. As there were investigated three times as many patients suffering from AMI compared to sepsis, the sepsis cohort could be underpowered for investigation of long-term mortality, but still clinical and laboratory markers differ significantly between AMI and septic patients with or without severe hyperglycemia.

Despite these limitations, we believe that our study is of value to under-strike the different implications of hyperglycemia in ICU patients admitted for either sepsis or AMI.

## Figures and Tables

**Table 1:** Baseline characteristics of the overall cohort, patients admitted for sepsis and patients admitted for AMI.

Parameter	Overall Cohort	Admitted for Sepsis	Admitted for MI	*p*-Value
age	69 (58–77)	68 (67–76)	69 (59–77)	0.18
male	61%	60%	62%	0.42
lactate (mmol/L)	2.69 ± 3.93	4.35 ± 4.94	2.10 ± 3.31	<0.001
glucose (mg/dL)	180 ± 72	195 ± 67	175 ± 73	<0.001
leucocytes (G/L)	12.43 ± 8.19	16.54±14.05	11.23±4.74	<0.001
(lowest) pO2 (kPa)	9.12 ± 2.12	5.35 ± 1.00	5.63 ± 1.92	0.005
pCO2 (kPa)	6.12 ± 1.85	6.76 ± 2.33	5.66 ± 1.38	<0.001
heart rate (bpm)	97 ± 23	118 ± 23	91 ± 20	<0.001
sodium (mmol/L)	140.02 ± 4.63	141.72 ± 7.14	139.65 ± 3.76	<0.001
potassium (mmol/L)	4.20 ± 0.54	4.27 ± 0.67	4.10 ± 0.50	<0.001
type 2 diabetes	13%	13%	13%	-
creatinine (µmol/L)	95 (78–95)	180 (112–297)	89 (76–116)	<0.001
ALAT (µmol/(l*s))	0.7 (0.4–1.3)	0.7 (0.3–1.4)	0.7 (0.5–1.2)	0.007
ASAT (µmol/(l*s))	1.6 (0.7–4.2)	1.0 (0.5–2.6)	2.1 (0.8–4.7)	<0.001
APACHE score	20 ± 10	26 ± 8	16 ± 8	<0.001
SAPS2 score	40 ± 20	54 ± 20	33 ± 16	<0.001

**Table 2:** In a Cox regression analysis, maximum glucose concentration on the day of admission was associated with mortality in the overall cohort and those patients admitted for AMI.

Group	HR	95% CI	*p*-Value
overall cohort	1.006	1.004–1.127	<0.001
admitted for sepsis	1.03	0.998–1.062	0.07
admitted for AMI	1.101	1.075–1.127	<0.001

**Table 3:** In the overall cohort, patients suffering from severe hyperglycemia (>200 mg/dL) were sicker (SAPS2 score 47 ± 20 vs. 39 ± 20; *p* < 0.001) and older (71 (62–78) vs. 68 (57–77) years; *p* < 0.001).

Parameter	<200 mg/dL	>200 mg/dL	*p*-Value
age	68 (57–77)	71 (63–78)	<0.001
male	60%	64%	0.04
lactate (mmol/L)	2.42 ± 3.12	4.05 ± 5.81	<0.001
glucose (mg/dL)	143 ± 30	267 ± 66	<0.001
leucocytes (G/L)	12.07 ± 8.49	15.10 ± 9.51	<0.001
(lowest) pO2 (kPa)	5.45 ± 1.44	5.26 ± 1.31	0.09
pCO2 (kPa)	5.88 ± 1.66	6.40 ± 2.16	<0.001
heart rate (bpm)	98 ± 23	107 ± 24	<0.001
sodium (mmol/L)	139.90 ± 4.41	140.67 ± 6.28	0.03
potassium (mmol/L)	4.12 ± 0.54	4.20 ± 0.64	0.07
type 2 diabetes	10%	25%	<0.001
creatinine (µmol/L)	95 (77–152)	120 (89–199)	<0.001
ALAT (µmol/(l*s))	0.7 (0.4–1.3)	0.8 (0.5–1.5)	<0.001
ASAT (µmol/(l*s))	1.4 (0.6–4.1)	1.8 (0.7–5.1)	<0.001
APACHE score	19 ± 9	24 ± 10	<0.001
SAPS2 score	39 ± 20	47 ± 20	<0.001
BMI (kg/m^2^)	27 ± 4	28 ± 5	<0.001

**Table 4:** Severe hyperglycemia was associated with adverse short-term outcome in the overall cohort (23% vs. 13%; *p* < 0.001) and patients admitted for AMI (15% vs. 5%; *p* < 0.001) but not for septic patients (39% vs. 40%; *p* = 0.48).

Group	HR	95% CI	*p*-Value	Glucose <200 mg/dL	Glucose >200 mg/dL
overall cohort	1.88	(1.46–2.43)	<0.001	13%	23%
admitted for sepsis	1.02	(0.71–1.47)	0.48	39%	40%
admitted for MI	3.02	(2.01–4.54)	<0.001	5%	15%

**Table 5:** Patients admitted for sepsis and suffering from severe hyperglycemia (>200 mg/dL) were of equal age (68 (55–76) vs. 68 (59–77) years; *p* = 0.21) and SAPS2 scores did not differ (52 ± 20 vs. 55 ± 20; *p* = 0.25).

Parameter	<200 mg/dL	>200 mg/dL	*p*-Value
age	68 (55–76)	68 (59–77)	0.21
male	61%	62%	0.85
lactate (mmol/L)	4.14 ± 4.76	4.75 ± 5.25	0.17
glucose (mg/dL)	156 ± 29	260 ± 62	<0.001
leucocytes (G/L)	15.22 ± 13.99	18.73 ± 13.95	0.006
(lowest) pO2 (kPa)	5.32 ± 0.95	5.39 ± 0.99	0.55
pCO2 (kPa)	6.60 ± 2.20	7.02 ± 2.51	0.04
heart rate (bpm)	118 ± 22	119 ± 23	0.65
sodium (mmol/L)	141.48 ± 6.96	142.14 ± 7.44	0.43
potassium (mmol/L)	4.27 ± 0.65	4.28 ± 0.70	0.92
type 2 diabetes	12%	17%	0.07
creatinine (µmol/L)	180 (112–292)	184 (114–317)	0.46
ALAT (µmol/(l*s))	0.7 (0.3–1.4)	0.7 (0.4–1.2)	0.69
ASAT (µmol/(l*s))	1.0 (0.4–2.5)	1.1 (0.5–2.9)	0.34
APACHE score	26 ± 8	28 ± 8	0.02
SAPS2 score	53 ± 20	55 ± 20	0.25

**Table 6:** In patients suffering from AMI and severe hyperglycemia (>200 mg/dL) were sicker (SAPS2 score 41 ± 16 vs. 32 ± 16; *p* < 0.001) and older (73 (65–79) vs. 68 (57–77) years; *p* < 0.001).

Parameter	<200 mg/dL	>200 mg/dL	*p*-Value
age	68 (57–77)	73 (65–79)	<0.001
male	59%	65%	0.03
lactate (mmol/L)	1.82 ± 1.94	3.65 ± 6.05	<0.001
glucose (mg/dL)	139 ± 30	272 ± 68	<0.001
leucocytes (G/L)	11.09 ± 5.23	13.22 ± 4.92	<0.001
(lowest) pO2 (kPa)	5.53 ± 1.71	5.12 ± 1.54	0.02
pCO2 (kPa)	5.54 ± 1.16	5.97 ± 1.76	<0.001
heart rate (bpm)	92 ± 20	101 ± 22	<0.001
sodium (mmol/L)	139.61 ± 3.65	140.04 ± 5.61	0.16
potassium (mmol/L)	4.11 ± 0.51	4.17 ± 0.61	0.12
type 2 diabetes	10%	29%	<0.001
Creatinine (µmol/L)	88 (75–114)	107 (86–155)	<0.001
ALAT (µmol/(l*s))	0.7 (0.4–1.3)	1.0 (0.58–1.6)	<0.001
ASAT (µmol/(l*s))	2.0 (0.7–4.9)	2.9 (1.1–6.2)	<0.001
APACHE score	16 ± 8	21 ± 8	<0.001
SAPS2 score	32 ± 16	41 ± 18	<0.001
BMI (kg/m^2^)	28 ± 4	29 ± 5	0.001

**Table 7:** In the overall cohort, patients with a medical history of diabetes (12%) were sicker in trend (SAPS2 score 43 ± 21 vs. 40 ± 20; *p* = 0.06) and older (72 (65–78) vs. 68 (57–76) years; *p* < 0.001).

Parameter	No T2DM	T2DM	*p*-Value
age	68 (57–76)	72 (65–78)	<0.001
male	61%	61%	0.9
lactate (mmol/L)	2.59 ± 3.17	3.27 ± 6.88	0.10
glucose (mg/dL)	175 ± 70	213 ± 76	<0.001
leucocytes (G/L)	12.43 ± 8.44	12.43 ± 6.46	0.99
(lowest) pO2 (kPa)	5.53 ± 1.68	5.46 ± 1.36	0.64
pCO2 (kPa)	6.02 ± 1.74	6.27 ± 2.38	0.06
heart rate (bpm)	97 ± 23	99 ± 24	0.23
sodium (mmol/L)	140.01 ± 4.55	14.10 ± 5.13	0.80
potassium (mmol/L)	4.13 ± 0.54	4.14 ± 0.54	0.65
BMI	28 ± 5	29 ± 4	<0.001
creatinine (µmol/L)	93 (77–146)	108 (86–174)	<0.001
ALAT (µmol/(l*s))	0.7 (0.4–1.3)	0.7 (0.4–1.2)	0.70
ASAT (µmol/(l*s))	1.7 (0.7–4.2)	1.4 (0.7–4.2)	0.85
APACHE score	20 ± 10	22 ± 10	0.02
SAPS2 score	40 ± 20	43 ± 21	0.06
intra-ICU survival	15%	15%	0.93
